# Oregon State Dental Society

**Published:** 1873-06

**Authors:** Wm. F. Thompson


### Proceedings of Societies.

### OREGON STATE DENTAL SOCIETY.

The several members of the dental profession throughout Oregon, having long felt the need of a State organization that would permit them to keep pace with and reap the same benefits as their Eastern brethren, met in Portland on March 29th, at 8 P. M., holding their meeting in Dr. Hatch's rooms, and organized a State Dental Society, electing the following officers: President, Dr. J. H. Hatch, Portland; Vice President, Dr. L. S. Skiff, Salem; Corresponding and Recording Secretary, Dr. Wm. F. Thompson; Portland; Treasurer, Dr. J. Welch, Portland.

After the organization was perfected the following persons signed the Constitution and By-Laws, becoming members of the society; Drs. J. H. Hatch, L. S. Skiff, Wm. F. Thompson, John Welch, George H. Chance, T. L. Nicklin, George W. Gray, J. R. Cardwell, and E. Biddle.

The meeting adjourned for refreshments.

After refreshment the society was called to order again, in Drs. Welch and Thompson's rooms, when a very interesting paper was read by Dr. Chance, upon gold-pointed instruments to be used as pluggers; also, explaining, in his opinion, their superior merit over steel.

After reading of the paper, the subject was taken up and fully discussed by the different members present.

Dr. Chance presented to several members an assortment of his instruments, to be tested by them and reported on at the next regular meeting.

A motion was then made and carried, that the next regular meeting of the Society be held at Dr. Skiff's rooms, Salem, on the last Wednesday in June. The subject for discussion at that meeting will be "Exposed Dental Pulps and their Treatment."

Dr. Gray was also appointed to prepare and read an essay before the Society at that time.

A motion was then made that a clinic be held on Monday, March 31st, at 10 A. M., and to adjourn until that time. Carried.

On motion, the Secretary was instructed to furnish copies of the proceedings for publication in the Daily Press of the city, and also to prepare and forward for publication a copy to the Dental .journals.

On motion the Society, adjourned until Monday, at 10 A. M.

### MORNING SESSION OF MONDAY.

The Society met pursuant to adjournment, March 31st, at the rooms of Drs. Welch and Thompson, Dr. J. H. Hatch, President, in the chair.

By suggestion of Dr. Thompson the office of Recording Secretary was declared vacant, and Dr. Cardwell elected to fill it for the ensuing year.

On motion, Dr. Thompson was invited to perform the clinic, whereupon the Doctor proceeded to the chair and made a difficult contour filling for Dr. Chance, which was done with superior skill, and much to the satisfaction of the Society. In the operation Dr. Thompson illustrated his manner of using the R. E. Carpenter engine and steel mallet.

On motion the meeting adjourned until 8 o'clock P. M.

EVENING SESSION.

Society met pursuant to adjournment, Dr. Hatch in the chair.

On motion, Drs. Chance, Gray and Thompson were appointed a committee to draft by-laws on the manner of becoming a member of this Society.

Subsequently the committee reported upon the subject, substantially requiring that " any dentist wishing to become a member of this society shall apply in writing, and a favorable vote of three-fourths of the members present shall be necessary to elect to membership.

The report of the committee was adopted.

Dr. Skiff made some remarks upon subject of the professional fees, and asked to hear from the others.

He was followed by Drs. Hatch, Gray, Glenn, Chance, and Cardwell.

The general view seemed to be favorable to first-class work, leaving the matter of fees as a secondary consideration, in order that an operator, when he went to the chair, should not be influenced by the class of the operation in consequence of preconceived ideas respecting the compensation. We should aim to have it distinctly understood by the patients, as well as by the members of this Society, that the prime object is the mutual advantage and benefit of all concerned.

Dr. Chance moved that the thanks of the Oregon State Dental Association should he hereby tendered to Drs. Welch and Thompson and Hatch for the use of rooms and other courtesies extended.

On motion of Dr. Gray, Drs. Hatch and Thompson were invited to operate in clinic at Dr. Skiff's office at the next meeting, to be held in Salem on the last Wednesday of June, 1873.

On motion the Association adjourned.

WM. F. THOMPSON, Secretary.

